# Differential expression of p38 MAPK α, β, γ, δ isoforms in nucleus pulposus modulates macrophage polarization in intervertebral disc degeneration

**DOI:** 10.1038/srep22182

**Published:** 2016-02-25

**Authors:** Chen Yang, Peng Cao, Yang Gao, Ming Wu, Yun Lin, Ye Tian, Wen Yuan

**Affiliations:** 1 Department of orthopedic Surgery, Changzheng Hospital, Shanghai 200003, China; 2 Kidney Institute, Department of Nephrology, Changzheng Hospital, Shanghai 200003, China; 3 National Key Laboratory of Medical Immunology & Institute of Immunology, Second Military Medical University, Shanghai 200433, China

## Abstract

P38MAPK mediates cytokine induced inflammation in nucleus pulposus (NP) cells and involves in multiple cellular processes which are related to intervertebral disc degeneration (IDD). The aim of this study was to investigate the expression, activation and function of p38 MAPK isoforms (α,β, γ and δ) in degenerative NP and the effect of p38 activation in NP cells on macrophage polarization. P38 α, β and δ isoforms are preferential expressed, whereas the p38γ isoform is absent in human NP tissue. LV-sh-p38α, sh-p38β transfection in NP cells significantly decreased the ADAMTS-4,-5, MMP-13,CCL3 expression and restored collagen-II and aggrecan expression upon IL-1β stimulation. As compared with p38α and p38β, p38δ exhibited an opposite effect on ADAMTS-4,-5, MMP-13 and aggrecan expression in NP cells. Furthermore, the production of GM-CSF and IFNγ which were trigged by p38α or p38β in NP cells induced macrophage polarization into M1 phenotype. Our finding indicates that p38 MAPK α, β and δ isoform are predominantly expressed and activated in IDD. P38 positive NP cells modulate macrophage polarization through the production of GM-CSF and IFNγ. Hence, Our study suggests that selectively targeting p38 isoforms could ameliorate the inflammation in IDD and regard IDD progression.

Low back pain (LBP) is one of the most popular health problems, and intervertebral disc degeneration (IDD) is thought to be a major cause for LBP^1^,^2^. Biotherapy is considered to be a potential therapeutic approach to prevent IDD when a molecular target is determined. Accumulating data suggest that inflammation plays a crucial role in the process of IDD^3^,^4^,^5^. Therefore, targeting inflammation may retard IDD and protect intervertebral disc tissue.

P38 Mitogen-Activated Protein Kinase (MAPK) is a proline-directed serine/threonine protein kinases which transduces signals from inflammation stimuli^6^,^7^. The p38MAPK family has four members: p38α(MAPK14), p38β(MAPK11), p38γ (MAPK12/ERK-6) and p38δ (MAPK13/SAPK4)^8^,^9^,^10^,^11^. The four isoforms share over 60% homology, but are selectively phosphorylated by distinct upstream kinases: Mitogen-Activated Protein Kinase Kinase 3 (MKK3), MKK4 or MKK6^12^,^13^. Among all p38 MAPK family members, p38α is well studied for its pro-inflammatory property, whereas the pro-inflammatory function of other isoforms is not completely understood^8^. The protective effect by p38MAPK inhibitor has been displayed in some chronic inflammatory diseases, such as rheumatoid arthritis (RA). However, in a phase III study, the side effect of p38MAPK inhibitor has been reported^14^,^15^,^16^. To reduce the toxicity of p38MAPK inhibitor, a new strategy has been proposed that p38 isoforms should be selectively targeted based on its activation and function status in certain pathological conditions.

P38 MAPK may play an important role in IDD. Pro-inflammatory cytokines are increased in IDD, and p38MAPK mediates cytokine induced expression of catabolic enzymes, such as ADAMTS-4,-5 and MMPs in nucleus pulposus (NP) cells^17^,^18^. Increased MMPs and ADAMTS-4,-5 could in turn degrade extra-cellular matrix (ECM) of NP, including Collagen-II (Col-II) and Aggrecan. Besides, p38MAPK is involved in multiple cellular processes such as apoptosis, autophagy, angiogenesis, and hypoxia which are related to IDD^19^,^20^. However, the activation of p38 *in vivo* and the differential expression/function of p38 isoforms in IDD are poorly understood.

Macrophages involve in many inflammatory diseases such as osteoarthritis (OA) and RA, however the role of macrophages in IDD is not known^21^,^22^,^23^,^24^. Our previous *in vitro* study have shown that upon cytokine stimulation, NP cells promotes macrophage migration by inducing the p38 MAPK mediated chemokine ligand 3 (CCL3) expression^25^. Macrophages can be classified into the classically activated (M1) or the alternatively activated (M2) phenotype. M1 macrophages which are marked with inducible nitric oxide synthase (iNOS) and CD86, produce high level of pro-inflammatory factors such as tumor necrosis factor-α (TNF-α), interleukin-6 (IL-6) and IL-12. Arginase-1 and CD206 are two markers for M2 Macrophages which produce immune-regulatory factors including IL-10 and CCL18^26^. Macrophages polarization status in IDD has not been reported. It has been shown that macrophage polarization can be modulated by cytokines such as interferon γ (IFNγ, granulocyte-macrophage colony-stimulating factor (GM-CSF), TNF-α, IL-1β, and monocyte chemotactic protein-1 (MCP-1) which could be expressed by p38MAPK activated NP cells^28^,^29^.

The aim of this study was to investigate the activation of p38 isoforms and polarization of macrophages in IDD. Moreover we intended to prove the hypothesis that p38 activation in NP cells triggers macrophage polarization.

## Results

### P38 activation in degenerated NP tissues

**Figure 1:**
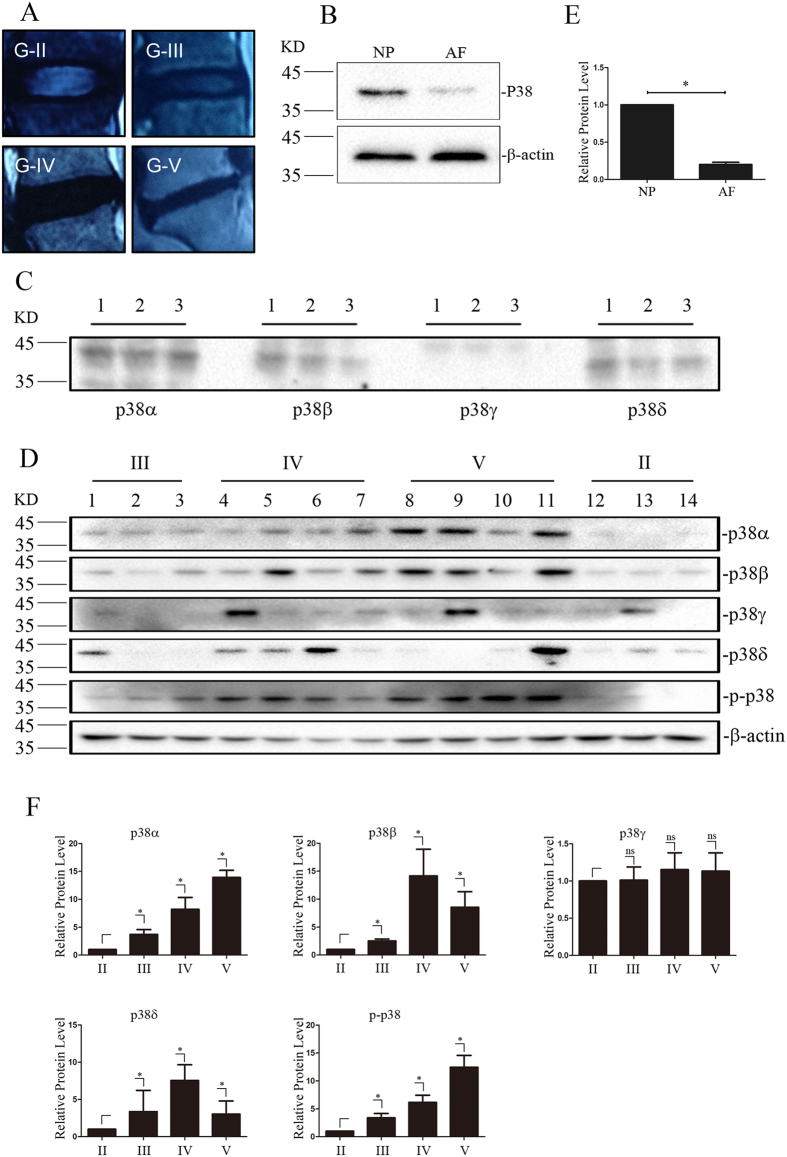
Differential expression of p38MAPK isoforms in degenerated nucleus pulposus tissues from patients with degenerative disc disease. (**A**) Representative images show grade II, grade III, grade IV, and grade V changes by MRI. (**B**,**E**) Western blot analysis of p38MAPK expression in degenerated NP and AF samples. Blots were was analysed by densitometry (n = 11, *P < 0.05). (**C**) Western blot analysis of pools of NP tissue stained with antibodies against the p38α, β, γ and δ (n = 11). (**D**,**F**) Western blot analysis of p38 α, β, γ and δ isoforms as well as phosphorylated p38 MAPK expression in degenerated nucleus pulposus samples of MRI grades II (n = 3), III (n = 3), IV (n = 4), and V (n = 4). Blots were was analysed by densitometry (*P < 0.05).

Figure 1A shows representative images classified as grade II (n = 3), grade III (n = 3), grade IV (n  =  4), grade V (n = 4) by MRI from 14 selected patients with degenerated nucleus pulposus and annulus fibrosis tissue. The expression of p38MAPK was significantly up-regulated in NP tissues compared to AF tissues as shown by western blot analysis of pooled extracts from 11 degenerated NPs tissues or 11 degenerated AFs tissues (Fig. 1B,E). The expression pattern of four p38 isoforms was further analyzed by western blot from these pooled tissues. Figure 1C shows that expression of p38α, β and δ can be detected but not p38γ. We next evaluated the expression pattern of p38 isoforms and phosphorylated p38 in NP extracts from 14 graded IDD patients. In comparison to normal NP tissues (grade II), the expression of p38α, β and p-p38 were remarkably increased in diseased tissues (Fig. 1D,F). And p38δ were only detected in half of IDD sample. The expression of p38α, β and p-p38 tended to increase with worsening of the disease (Fig. 1D,F). Whereas all of 14 NP tissue displayed very low or almost no p38γ expression (Fig. 1D,F).

### Inflammatory cell infiltration in degenerated NP tissues

To study inflammatory cell infiltration in NP tissues, we use several cell type specific markers (Table S1) to stain 300 samples from 278 patients, including grade II (n = 15), grade III (n = 93), grade IV (n = 108), grade V (n = 84) (Table S1). None of samples shows positive staining for lymphocytes (CD20, CD45RO, CD4, CD8) or dendritic cells (CD1a, S100), however almost all of degenerated samples (grade III, IV, V) and 2 of 15 normal specimens (grade II) shows positive staining for CD45 or CD68 (macrophage markers). And approximately 70% of degenerated NP tissues show CD11b (macrophage marker) positive staining, whereas all of normal samples are CD11b negative.

**Figure 2:**
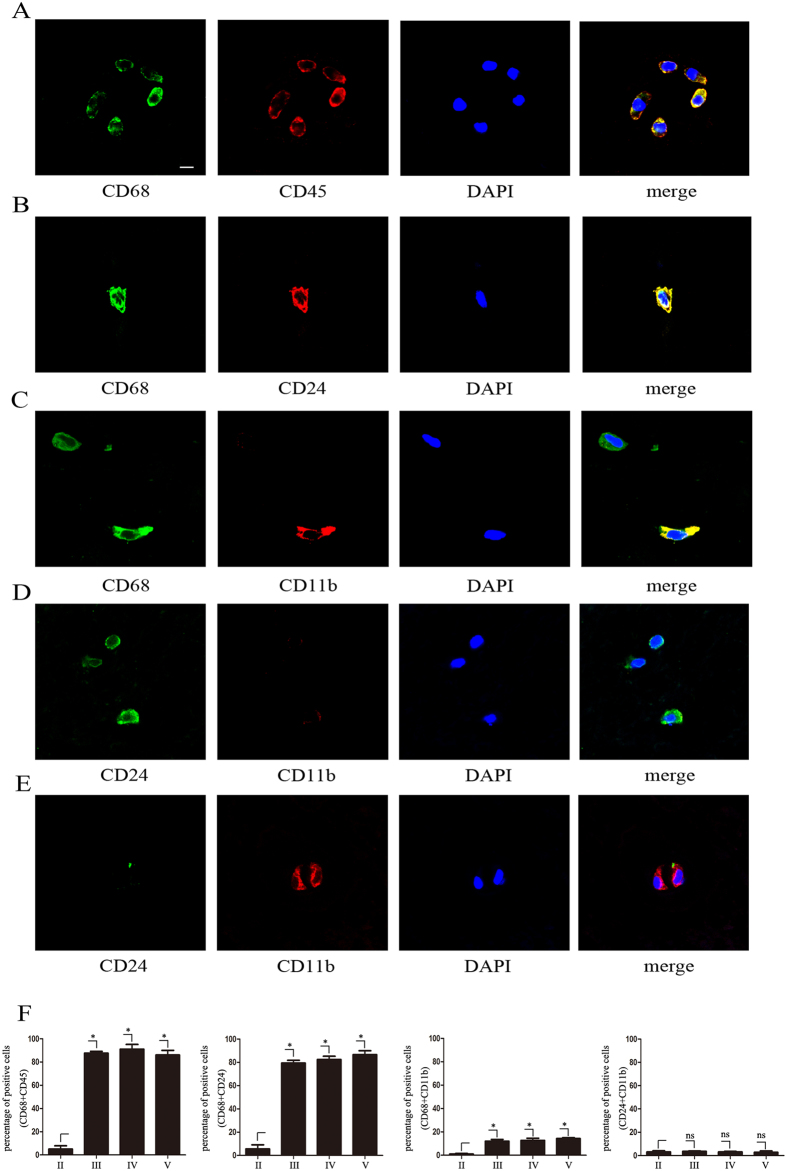
Immunofluorescence staining of NP tissue with antibodies against cell Immunophenotype. (**A**) cells in NP were immunolabelled for CD68 (green) and CD45 (red), cell nucleus were blue counterstained using DAPI. (**B**) In degenerated NP, almost all NP cells (CD24, red) were co-stained with CD68 (green). (**C**) all cells express CD68 positive, but CD11b immunopositivity were only present in a few cells. (**D**,**E**) cells in degenerated NPs, showed either CD24 positive or CD11 positive. (**F)** percentage of cells positive for respective markers in the NP of IDD (Grade III, IV, V) compared with control (Grade II) (In these experiments, all 300 human samples were used, *P < 0.05.) Bar = 50 μm.

To further characterize the type of infiltrated cells in degenerated NP, double-labeling staining with macrophage markers (CD45, CD68 or CD11b) and NP cell specific marker (CD24) were carried out. 88% of cells in grade III samples, 91% of cells in grade IV and 84% of cells in grade V showed double positive for CD45 and CD68, (Fig. 2A,F), whereas only 5% cells in grade II samples stained positive. Interestingly, 80% of cells in grade III samples, 86% of cells in grade IV and 91% of cells in grade V shows both CD24 and CD68 positive (Fig. 2B,F), indicating CD68 is not specific for macrophages in this study. Moreover, 15%, 18% and 20% of cells in grade III, IV and V respectively are double positive for CD11b and CD68, whereas only 2% double positive cells are present in grade II (Fig. 2C,F). Our data suggests that only CD11b is specific as a macrophage marker in NP tissues. To better differentiate NP cells and macrophages in degenerated NP tissues, CD24 and CD11b double-labeling were performed. As expected, cells in degenerated NP tissues showed either CD24 positive or CD11b positive, and few cells are double positive for both CD24 and CD11b (Fig. 2D–F).

Collectively, our data suggests that macrophage is the only type of infiltrated inflammatory cells in degenerated NP tissue.

### Differential expression of p38 MAPK isoforms in NP cells and Macrophage in degenerated NP tissues

**Figure 3:**
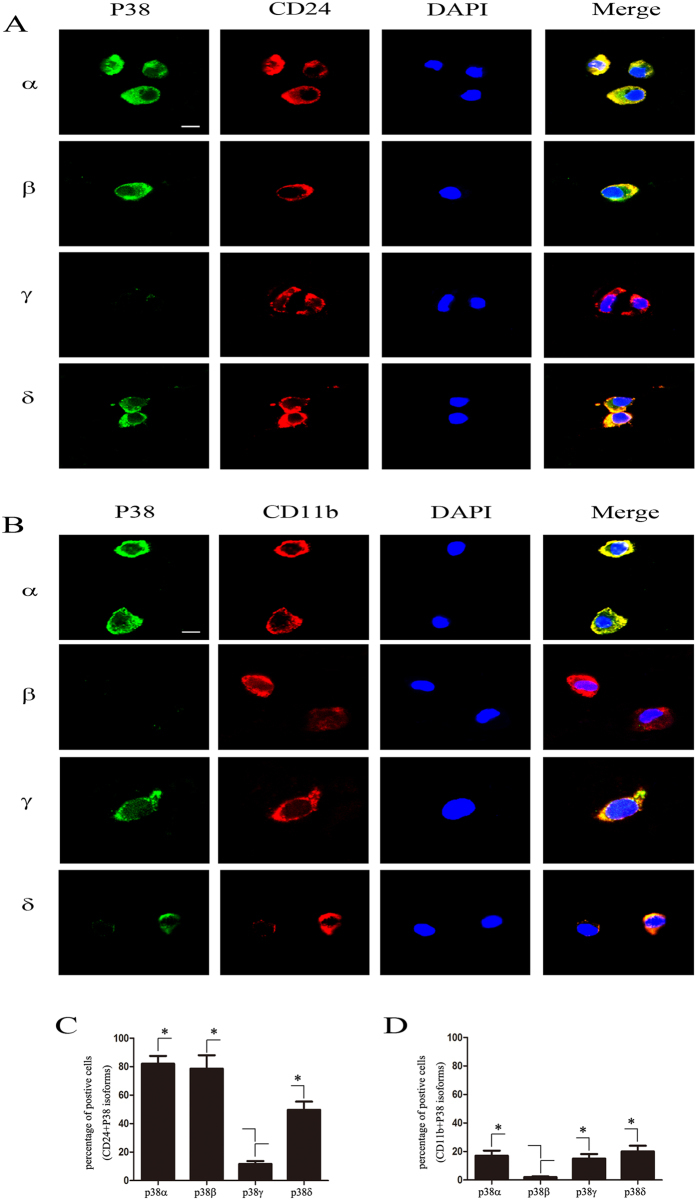
Expression of p38 isoforms in degenerated NP *in situ*. (**A**) NP cells, marked with CD24 (red), express p38 α, β, δ but not γ isoforms (green) analyzed by Immunofluorescence staining. Bar  =  50 μm. (**B**) a few macrophage in degenerated NP, marked with CD11b (red), express p38 α, γ, δ but not β isoforms (green). Bar  =  5 μm. (**C**) percentage of CD24 postive NP cells express p38 α or β or γ or δ in degenerated NP samples (n = 150, *P < 0.05). (**D**) percentage of CD11b positive cells co-stained with p38 α or β or γ or δ in degenerated NP samples (n = 150, *P < 0.05).

We next study the expression pattern of p38 MAPK isoforms in NP tissues by double-labeling immunofluorescence staining of 150 degenerated NP samples (grade III (n = 50), grade IV (n = 50), grade V (n = 50)). 83% and 78% of cells co-express CD24 with α or β isoforms respectively in degenerated tissue, whereas the p38δ isoform co-stained with CD24 was detected in 56% of cells in degenerated tissue (Fig. 3A,C). The double staining of CD24 with γ isoform was observed in only 9% cells in degenerative samples (Fig. 3A,C). Interestingly, almost all of CD11b positive macrophages (20% of total number of cells) expressed α, γ and δ isoform in degenerative samples (Fig. 3B,D). Together, these data revealed the differential expression pattern of p38 isoforms in degenerated NP tissue.

### Differential activation of p38 MAPK isoforms in NP cells of degenerated IVD

**Figure 4:**
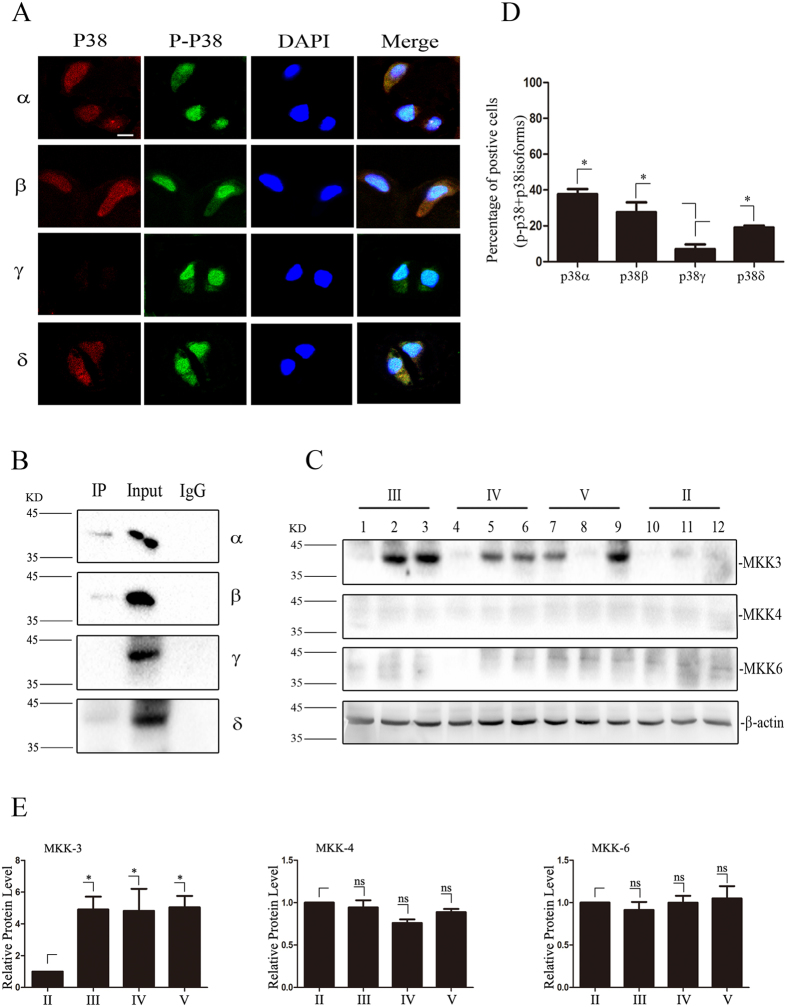
Activation of p38 isoforms in degenerated NP tissue. (**A**) Immunofluorescence double-labeling of phosphorylated p38 MAPK (green) and p38 MAPK α, β, γ and δ isoforms (red) in the degenerated NP tissue. Phosphorylated p38 that are double-labeled with the α, β and δ isoforms. Bar  =  50 μm. (**B**) Pooled extracts of NP tissue from IDD patients was subjected to immunoprecipitation with an antibody against phosphorylated p38 MAPK. The IP group, Input group and IgG group, were then stained for each p38 MAPK isoform (n = 11). (**C**,**E**) Western blot analysis of upstream kinases, MKK3, 4, 6 in degenerated NP tissue of MRI grades II (n = 3), III (n = 3), IV (n = 3), and V (n = 3). Blots were was analysed by densitometry. (*P < 0.05) (**D**) Quantification of the percent of cells expressing each p38 MAPK isoform that also labeled with phosphorylated p38 MAPK in degenerated NP samples (n = 150, *P < 0.05).

To investigate the activation status of different p38 isoforms in NP tissues, immunofluorescence double-labeling for p38MAPK isoforms and phosphorylated p38 MAPK were performed in over 150 samples. Phosphorylated p38 MAPK was co-localized with α or β isoform in 38% and 33% cells of degenerated NP tissues respectively, but only co-stained with δ isoform in 24% cells of degenerated NP tissues, whereas there was only less than 5% cells co-stained with γ isoform in any samples (Fig. 4A,D).

To further confirm these findings, we pulled down four p38MAPK isoforms through isoform-specific antibodies from pooled protein extracts of 11 degenerated NP specimens and then probed with phosphorylated p38MAPK antibodies. As expected, phosphorylated p38MAPK can only be detected in pulled-down proteins by p38α, β or δ isoform antibodies (Fig. 4B). Moreover, γ isoform could not be detected in the precipitates (Fig. 4B).

In order to study the pathway upstream of p38MAPK, we analyzed the expression of 3 type of MAPK kinases (MKK-3,-4,-6). As shown in Fig. 4C,E, only MKK-3 was up-regulated in degenerated samples as compared with grade II samples. The expression of MKK-4 or MKK-6 was not changed in all samples (Fig. 4C,E).

Altogether these results indicates that p38α, β and δ were preferential activated during the process of IDD.

### Functions of p38MAPK isoforms in human NP cells

**Figure 5:**
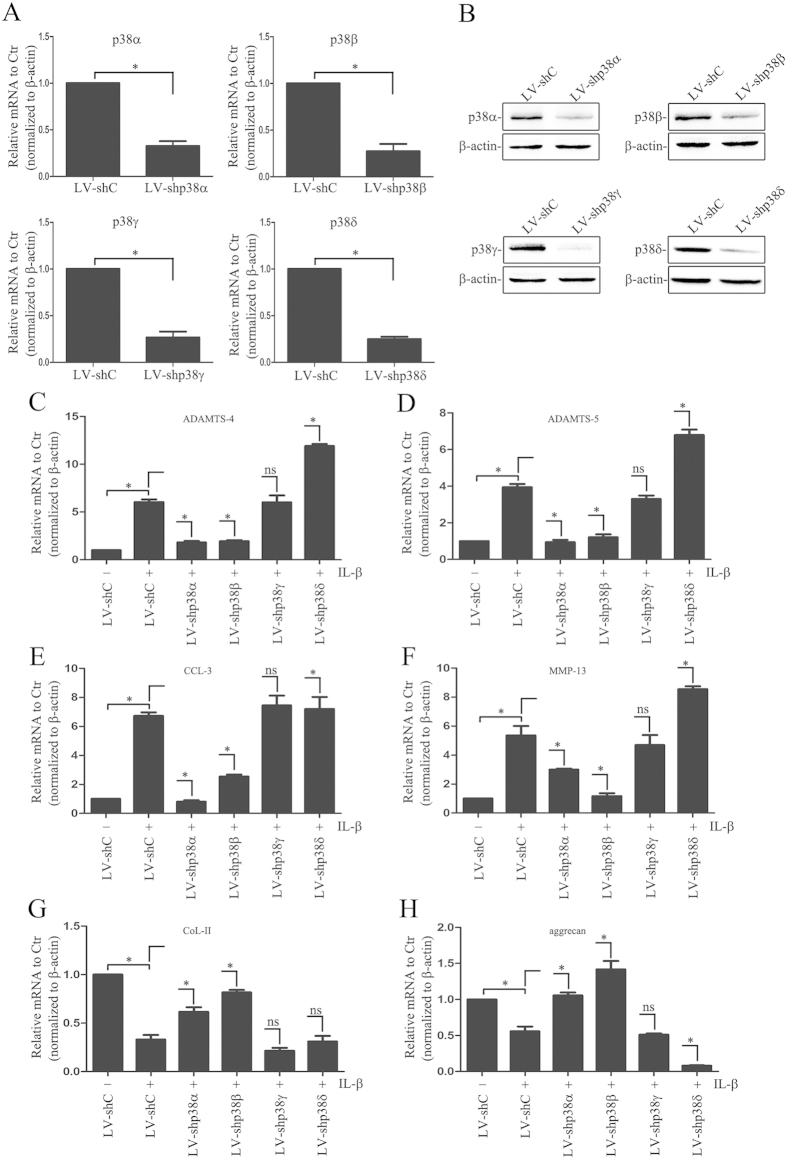
Real-time RT-PCR analysis of cells transfected with a lentivirus expressing control shRNA (sh-C), sh-p38α, sh-p38β, sh-p38γ or sh-p38δ after IL-1β treatment for 24h. (**A**,**B**) mRNA and protein expression of p38α, p38β, p38γ or p38δ in cells transfected with a lentivirus expressing control shRNA (sh-C), sh-p38α, sh-p38β, sh-p38γ or sh-p38δ (n = 3, respectively). (**C**–**F**) the mRNA expression of ADAMTS-4,-5, MMP-13 and CCL3 were analyzed (n = 3, respectively). (**G**,**H**) the mRNA expression of collagen II and aggrecan were analyzed (n = 3, respectively). Data show the mean ± SEM from 3 independent experiments. *P < 0.05.

We next investigate the pro-inflammatory role of p38MAPK isoforms in IDD. After 24h IL-1β treatment, the expression of p38MAPK isoforms in human NP cells were silenced with lentivirus expressing sh-p38α, β, γ or δ respectively, and several IDD-related genes were examined in the mRNA level. The transfection efficiency was assessed by q-PCR and WB. As shown in Fig. 5A,B, there was a significant decrease in mRNA and protein levels of p38α, β, γ or δ in cells transfected with sh-p38α or β or γ or δ respectively, in comparison to cells transfected with control shRNA. Figure 5C–F shows that IL-1β induced up-regulation of ADAMTS-4,-5, MMP-13 or CCL3 mRNA expression. Suppression of p38α or β but not γ isoform significantly decrease the expression of ADAMTS-4,-5, MMP-13 and CCL3 induced by IL-1β (Fig. 5C–F). Surprisingly, knock down p38δ significantly up-regulated ADAMTS-4, -5 or MMP-13 mRNA expression (Fig. 5C–F). In addition, IL-1β decreased collagen-II and aggrecan mRNA expression. The sh-p38α or β restored the expression of collagen-II and aggrecan which were suppressed by IL-1β, whereas sh-p38γ or δ had no effect on collagen-II expression (Fig. 5). Interestingly, only sh-p38δ but not sh-p38γ resulted in a small but significant suppression on aggrecan mRNA expression.

### Preferential expression and activation of p38 α, β and δ in NP cells associate with macrophage polarization in IDD

**Figure 6:**
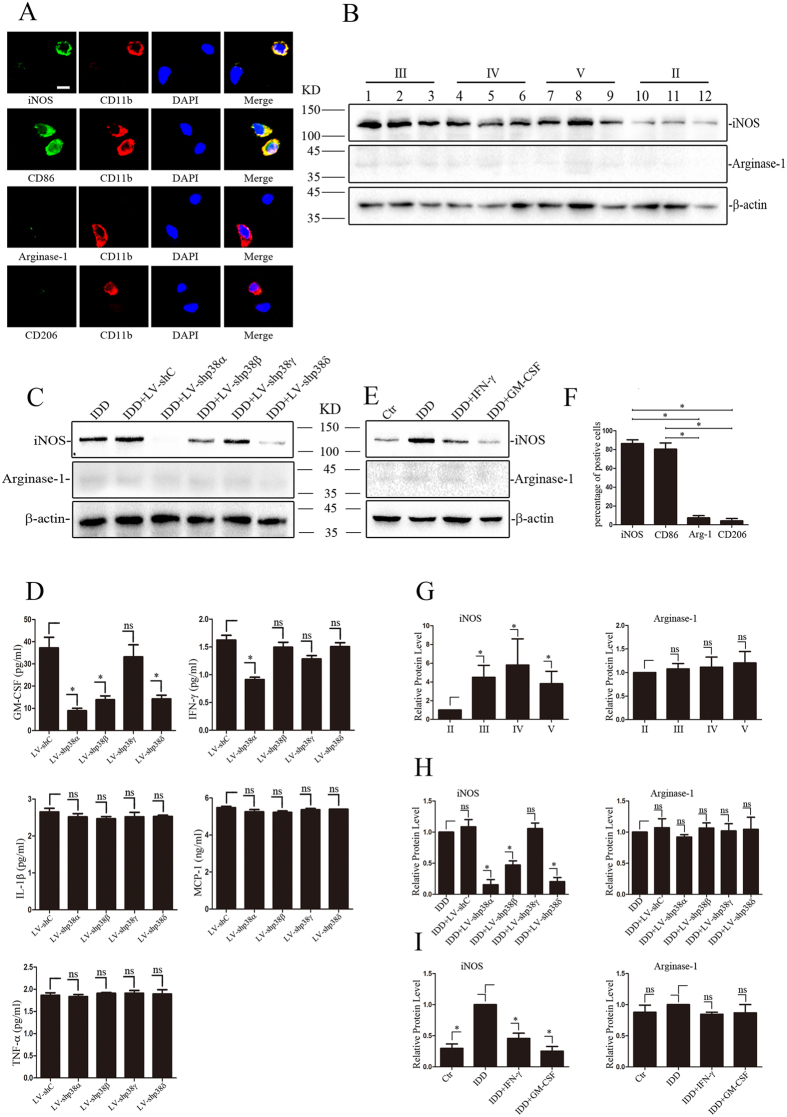
Macrophage M1 polarization in degenerated NP tissue. (**A**) Immunofluorescence double-labeling of iNOS or CD86 (green) and CD11b (red) in the degenerated NP tissue. Macrophage in degenerated NP tissue displayed positive CD86 and iNOS staining, which were M1 marker. Moreover, M2 marker (Arginase-1 and CD206) were negative. Bar = 50 μm. (**B**,**G**) Western blot analysis of macrophage treated with CM of primary NP cells from degenerated nucleus pulposus samples of MRI grades II (n = 3), III (n = 3), IV (n = 3), and V (n = 3). The M1 marker iNOS and M2 marker Arginase-1 were analyzed. Blots were was analysed by densitometry (*P < 0.05). (**C**,**H**) Macrophage treated with CM of primary human NP cell (grade III) was transfected with lentivirus expressing sh-p38α or β or γ or δ or sh-C immediately. The M1 marker iNOS and M2 marker Arginase-1were analyzed. Blots were was analysed by densitometry (n = 3, respectively, *P < 0.05). (**D**) flow cytometric analysis of cytokines in CM from primary human NP cells (grade III) which were transfected with lentivirus expressing sh-p38α or β or γ or δ or sh-C immediately. The expression of GM-CSF, IFNγ, MCP-1, IL-1β and TNF-α were analyzed (n = 3, respectively, *P < 0.05). (**E**,**I**) macrophage were treated with CM including neutralization antibodies against GM-CSF or IFNγ. The M1 marker iNOS and M2 marker Arginase-1were analyzed. Grade II sample served as a normal control. Blots were was analysed by densitometry (n = 3, respectively, *P < 0.05). (**F**) Quantification of the percent of CD11b positive cells expressing M1 marker(iNOS and CD86) or M2 marker(Arginase-1 and CD206) (n = 150, *P < 0.05).

To investigate the macrophage polarization status in degenerated samples, all of 285 IDD samples were detected by double-labeling immunofluorescence. Figure 6A shows that 84% and 80% of CD11b positive cells also strongly express the M1 macrophage marker iNOS or CD86 respectively, whereas the M2 marker Arginase-1 and CD206 can only be detected in only 5% and 7% of CD11b positive cells, suggesting macrophages infiltrated in NP tissues inclined to M1 polarization.

To study whether NP cells influence macrophage M1 polarization, conditioned-media (CM) of human NP cells from various degree of degenerated samples (n = 12) were collected and cultured for 3 days, then treated RAW264.7 macrophages. Compared to CM of normal NP cell (grade II), macrophage treated with CM of degenerated NP cells, especially grade III and grade IV, express remarkably higher iNOS, whereas all samples showed almost no Arginase-1 expression (Fig. 6B,G).

To further investigate if p38 isoforms in NP cells impact macrophage polarization, primary human NP cells (grade III) were transfected with lentivirus expressing sh-p38α, β, γ, δ or control shRNA immediately after isolation and cultured for 3 days, then CM were used to treat macrophages. Compared with control cells, iNOS expression was completely suppressed by CM from cells transfected with sh-p38α and partially suppressed by CM from cells transfected with sh-p38β or δ, whereas CM of cells transfected with sh-p38γ had no effect on macrophage polarization (Fig. 6C,H). In addition, all of samples show no Arginase-1 expression (Fig. 6C,H).

Furthermore, with flow cytometric bead array we examined the expression of cytokines in CM, which might be linked to M1 polarization. As is shown Fig. 6D, the suppression of p38α, β, γ or δ had no effect on the expression of TNF-α, IL-1β and MCP-1, whereas knockdown of p38α induced a small but significant decrease in IFNγ production. Notably, knockdown of p38α, β or δ significantly suppressed the expression of GM-CSF (Fig. 6D). In addition, after primary human NP cells were isolated and cultured for 2 days, anti- IFNγ or anti-GM-CSF neutralizing antibodies were added respectively for another 24h culture, then the CM were collected to treat macrophages. Western blot analysis shows that blocking IFNγ partially decreased the expression of iNOS, and neutralizing GM-CSF almost completely abolished the expression of iNOS (Fig. 6E,I). Again, the expression of Arginase-1 could not be detected in any samples (Fig. 6E,I).

## Discusion

In this study we discovered: 1) p38 MAPK is up-regulated and activated in the degenerated disc; 2) α, β or δ isoform of p38 MAPK predominantly present and activate in the degenerated disc; 3) macrophages are the only type of inflammatory cells infiltrated in the degenerated disc and correlated with disease progression; 4) the infiltrated macrophages are differentiated into M1 phenotype which is triggered by p38 positive NP cell released GM-CSF and IFNγ.

It has been shown that p38 MAPK plays a central role in the inflammatory response *in vitro* in NP cells^29^,^30^,^31^. Micro-vascular system has been found to invade NP even at early stage of IDD suggesting a pro-inflammatory micro-environment may exist at the early stage of IDD^32^,^33^, therefore we hypothesized that p38 play a pro-inflammatory role in disease progression of disc degeneration. Indeed, our study showed that p38 is over-expressed in NP compared to AF. Furthermore we showed that p38 and its activated form are positively correlated with the grade of disc degeneration. Moreover we found that differentiation of macrophages is triggered by inflammatory factors derived from p38 activated NP cells.

P38 isoforms expression are tissue and cell type dependent, suggesting that p38 exerts different functions through its different isoforms^34^. In this study we found that p38α, p38β and p38δ other than p38γ isoforms were preferentially expressed and activated in degenerated NP. And we confirmed that MKK3 which could activate p38α, β, and δ was the major upstream kinase of p38MAPK in NP, rather than MKK6 which activates all four p38 isoforms or MKK4 which activates p38α and δ. Moreover the expression of α and β but not δ isoforms correlated positively with the grade of degeneration. Furthermore specific knock-down α and β isoforms reversed IL-1β induced abnormal expression of degenerative markers such as ADAMTS-4,-5, MMP-3, CCL3, collagen II and aggrecan. Surprisingly, knock-down of p38δ enhanced the abnormal expression of degenerative markers such as ADAMTS-4,-5, MMP-3, and decreased aggrecan expression in NP cells in response to IL-1β stimulation. Coincidentally, Hiyama *et al.* also found the protective role of p38δ isoform in NP cell^35^. Moreover, opposite effects between p38α isoform and p38δ isoform had been reported before^36^. Taken together, we speculated that p38δ isoform may exert protective effect for IDD though antagonizing the effect of p38α isoform. All these findings suggest that specifically inhibiting or activating the isoform of p38MAPK could be used to prevent the disc degeneration.

A large body of evidence indicate that numerous inflammatory cells, such as monocytes/macrophages, lymphocytes and granulocytes are infiltrated in herniated disc^37^,^38^,^39^, however whether and which type of inflammatory cells exist in degenerated but contained samples is not known. To our knowledge we are the first group finding that macrophages are the only type of inflammatory cells infiltrated in the contained NP. Our result suggests that role of macrophage in contained NP more likely to be a normal inflammatory response to tissue injury rather than an antigen-specific immune response which is widely considered in regression of herniated disc where lymphocytes exist. We also showed that the number of macrophages positively correlated with the grade of IDD, suggesting a detrimental role of macrophages in disease progression of IDD.

Once monocytes migrate to injury tissues, they will differentiate into different macrophage phenotypes in response to the local micro-environment^26^. Macrophages exert either pro-inflammatory functions through M1-phenotype macrophages to clean up foreign invaders/damaged cells or exert proliferative functions through M2-phenotype macrophages to repair injured tissues^26^,^40^. We found that macrophages differentiated into M1 phenotype in degenerated discs. It has been shown that pro-inflammatory macrophages participate in disc degeneration through digesting ECM^41^,^42^. Our data suggest that macrophages drive disease progression of IDD through its pro-inflammatory properties.

It is know that macrophages differentiate into M1 phenotype in response to the challenge of inflammatory factors such as GM-CSF, IFNγ, MCP-1,TNF-α and IL-1β. Interestingly, p38 could modulate local mico-environment through regulating the expression of these inflammatory factors^28^,^40^,^43^. Therefore we hypothesized that activation of p38 in NP cells trigger macrophage differentiation through modulating local pro-inflammatory micro-environment. Indeed, we found that: 1) CM collected from primary human degenerated NP cells triggered M1 phenotype conversion; 2) blockage of p38α, β or δ in primary NP cells inhibited M1 phenotype differentiation; 3) blockage of p38 isoforms reduced the production of GM-CSF and IFNγ by primary NP cells; 4) neutralization of GM-CSF or IFNγ in CM prevent M1 macrophage differentiation. Our data suggest that p38 activation in NP cells triggered microphage M1 phenotype differentiation through creating a pro-inflammatory micro-environment by producing GM-CSF and IFNγ.

In summary, we found a novel role of p38 in disease progression of IDD. Selectively blocking or activating p38 isoforms could be a new strategy to prevent human disc degeneration.

## Materials and Methods

### Reagents and plasmids

The anti-p38α and p-p38 used in western blot (WB) or immunoprecipitation (IP) or immunofluorescence (IF), and anti-p38β, anti-p38γ, anti-p38δ used in WB or IP, and anti-β-actin, anti-MKK-3, anti-MKK-4, anti-MKK-6 used in WB were purchased from CST. The anti-p38βand CD206 were purchased from ST, and anti-p38γ and anti-p38δ were from RD, the anti-CD68, anti-CD45, anti-CD24, anti-CD11b, anti-iNOS and anti-Arginase-1 were obtained from Abcam were used in IF. Anti-IFNγ and anti-GM-CSF uesd in neutralising assay were purchased from Abcam. BD^TM^ Flow Cytometric Bead (CBA) Array flex set (Human IFNγ GM-CSF, TNF-α, IL-1β, MCP-1 flex set) was purchased from BD Biosciences. Plasmids short hairpin RNA for p38α, p38β p38γ and p38δ in a lentiviral GV118 vector were obtained from Genechem (Genechem Shanghai, China). IL-1β was purchased from PeproTech.

### Human tissue collection and grading

Informed consent was obtained from all subjects before their participation in the study. The study was approved by the ethical committees of Changzheng Hospital (Shanghai, China). All the subsequent research analyses were carried out in accordance with the approved guidelines. Human lumbar IVD tissues were obtained from patients’ undergoing surgery. All thee samples were graded based on Pfirrmann’s grading system. Detail information of 300 IVD tissues from 278 patients were described in Table S1. NP tissues were divided into 2 sections, one of which was fixed in 10% neutral buffered formalin and further processed for immunofluorescence staining. Others were used for western blot analysis and primary cell culture.

### Cell culture and collection of conditioned medium

Human NP cells were isolated from IVD of patients with IDD using the method previously described by Risbud *et al.* The isolated NP cells were maintained in Dulbecco’s modified Eagle’s medium with 10% fetal bovine serum supplemented with antibiotics. To investigate macrophage polarization, the conditioned medium (CM) of human primary NP cells from patients with IDD were collected. RAW264.7 cell line was purchased from ATCC.

### Protein extraction and immunoprecipitation

Human NP tissues protein extraction: human NP tissues were immediately frozen in liquid nitrogen after isolation. Then, frozen NP tissues were mechanically homogenized at 4 °C in RIPA lysis buffer contained protease inhibitor cocktail (Piemrce). Cell Protein extraction: RAW264.7 cells were immediately placed on ice and then washed using ice-cold PBS three times. Then cells were spall at 4 °C in RIPA lysis buffer including protease inhibitor cocktail (Piemrce). Immunoprecipitation was performed using Protein A/G PLUS-Agarose beads (Santa Cruz) according to the manufacturer’s standard protocol.

### Western blotting

Total protein was resolved on 10% sodium dodecyl sulfate-polyacrylamide gels and transferred to nitrocellulose membrane by electroblotting (Bio-Rad). The membranes were blocked using 5% nonfat dry milk in TBST (50 mM Tris, pH 7.6, 150 mM NaCl, 0.1% Tween 20) and then incubated overnight at 4 °C in 3% nonfat dry milk in TBST with antibodies against p38 MAPK isoforms, phosphorylated p38MAPK, iNOS, Arginase-1, MKK-3, MKK-4, MKK-6 or anti- β-tubulin. Data are from three independent experiments.

### Double immunofluorescence staining of frozen specimens

Specimens were snap-frozen and cut in 8 μm transversally with a cryostat microtome. The frozen sections were fixed in methanol at −20 °C for 30 min. Then, samples were incubated for 10 min with PBS containing 0.25% Triton X-100 for permeabilization. Before treatment with the primary antibody, nonspecific binding was blocked by treatment with 1% bovine serum albumin. Specimens were simultaneously incubated with 2 different source primary antibodies overnight at 4 °C. After washing with PBS three times, specimens were incubated with two corresponding secondary antibody for 30 min in the dark at room temperature. For DNA counterstaining, samples were incubated in 0.1 μg/ml DAPI (4′-6-diamid-ino-2-phenylindole) for 1 min then washed using PBS. Images were acquired with a Leica confocal microscopy (Wetzlar, Germany). Quantitative analysis was performed by two pathologists who were blinded to the clinical data. The positively stained cells and cell clusters were counted as a fraction of the area.

### Real-time quantitative reverse transcription polymerase chain reaction (Q-PCR)

Total RNA in NP cells was isolated using TRIzol® Reagent (Ambion Invitrogen, Carlsbad, CA,USA) according to the manufacturer’s protocol. RNA concentrations were measured using a NanoDrop instrument (NanoDrop, Wilmington, DE, USA). Reverse transcription to cDNA was performed using a High-Capacity cDNA Archive Kit (ABI, Foster City, CA, USA). Primers were designed and purchased from Sangon (Sangon, Shanghai, China; see Supporting information, Table S2). The value of gene expression was determined after normalization to β-actin. All gene Q-PCR, including β–actin controls, were run in triplicate in a GeneAmp PCR 9700 Thermocycler (ABI). The relative amounts of mRNA were calculated using the comparative Ct (2^−^ΔΔ^Ct^) method.

### Flow Cytometric Bead Array flex set

Cytokines GM-CSF, INFγ, MCP-1, IL-1β and TNF-α were detected using the BD CBA Human Soluble Protein Flex Sets system (Becton Dickinson, Heidelberg, Germany) according to the manufacturer’s instructions. This system uses the sensitivity of amplified fluorescence detection with flow cytometry to measure a soluble analyte. BD CBA Human Soluble Protein Flex Set capture bead is a single bead population with distinct fluorescence intensity and is coated with a capture antibody specific for a soluble protein. Samples were analyzed on a FACSCanto (Becton Dickinson).

### Statistical Analysis

All the statistical analyses were performed using SPSS version 17.0 software. For comparisons, Students’ t test or one-way analysis of variance (ANOVA) followed by the Turkey’s post-hoc test or Kruskal-Wallis test were performed, as appropriate. P < 0.05 was considered significant.

## Additional Information

**How to cite this article**: Yang, C. *et al.* Differential expression of p38 MAPK α, β, γ, δ isoforms in nucleus pulposus modulates macrophage polarization in intervertebral disc degeneration. *Sci. Rep.* **6**, 22182; doi: 10.1038/srep22182 (2016).

## Supplementary Material

Supplementary Information
