# Supplementary material for: Differential expression of p38 MAPK α, β, γ, δ isoforms in nucleus pulposus modulates macrophage polarization in intervertebral disc degeneration
Source: Sci Rep. 2016 Feb 25;6:22182. doi: 10.1038/srep22182 (PMC4766431; doi:10.1038/srep22182)
Supplement: Supplementary Information [file srep22182-s1.docx]

**Differential expression of p38MAPK α,** **β, γ, δ isoforms in nucleus pulposus modulates macrophage polarization in** [**intervertebral**](app:ds:intervertebral) [**disc**](app:ds:disc) **degeneration**

**Short running title: p38 isoforms in human nucleus pulposus**

Chen Yang^1†^, Peng Cao^1†^, Yang Gao^1†^, Ming Wu^2^, Yun Lin^3^, Ye Tian^1*^ and Wen Yuan^1*^

^1^ Department of orthopedic Surgery, Changzheng Hospital, Shanghai 200003, China

^2^ Kidney Institute, Department of Nephrology, Changzheng Hospital, Shanghai 200003, China

^3^ National Key Laboratory of Medical Immunology & Institute of Immunology, Second Military Medical University, Shanghai 200433, China

^†^These authors contributed equally to this study.

* Correspondence and requests for materials should be addressed to Ye Tian (email: [hrs918@163.com](mailto:hrs918@163.com)) or Wen Yuan (email:[yuanwenczspine@163.com](mailto:yuanwenczspine@163.com)).

**TableS1. Immunophenotype of the Inflammatory Cell Infiltrates**

| Case | Age | Gender | Site | Grade | CD20 | CD45RO | CD4 | CD8 | CD1a | S100 | CD68 | CD45 | CD11b |
| --- | --- | --- | --- | --- | --- | --- | --- | --- | --- | --- | --- | --- | --- |
| 1 | 22 | M | L3/4 | II | - | - | - | - | - | - | - | - | - |
| 2 | 15 | M | L2/3 | II | - | - | - | - | - | - | + | + | - |
| 3 | 18 | M | T12/L1 | II | - | - | - | - | - | - | - | - | - |
| 4 | 23 | F | L2/3 | II | - | - | - | - | - | - | - | - | - |
|  |  |  | L3/4 | II | - | - | - | - | - | - | - | - | - |
| 5 | 31 | F | L4/5 | II | - | - | - | - | - | - | - | - | - |
| 6 | 17 | M | L4/5 | II | - | - | - | - | - | - | - | - | - |
| 7 | 18 | M | L3/4 | II | - | - | - | - | - | - | - | - | - |
|  |  |  | L4/5 | II | - | - | - | - | - | - | - | - | - |
| 8 | 15 | M | T12/L1 | II | - | - | - | - | - | - | + + | + + | - |
| 9 | 20 | F | T11/12 | II | - | - | - | - | - | - | - | - | - |
| 10 | 16 | F | L4/5 | II | - | - | - | - | - | - | - | - | - |
| 11 | 22 | M | L5/S1 | II | - | - | - | - | - | - | - | - | - |
| 12 | 25 | F | L3/4 | II | - | - | - | - | - | - | - | - | - |
| 13 | 19 | F | L3/4 | II | - | - | - | - | - | - | - | - | - |
| 14 | 48 | M | L4/5 | III | - | - | - | - | - | - | + + | + + | + |
| 15 | 69 | M | L4/5 | III | - | - | - | - | - | - | + + | + + | + |
| 16 | 55 | M | L5/S1 | III | - | - | - | - | - | - | + + | + + | + |
| 17 | 43 | M | L4/5 | III | - | - | - | - | - | - | + + | + + | + |
| 18 | 49 | M | L4/5 | III | - | - | - | - | - | - | + + | + + | + |
| 19 | 46 | M | L4/5 | III | - | - | - | - | - | - | + + | + + | + |
| 20 | 79 | F | L4/5 | III | - | - | - | - | - | - | + + | + + | + |
| 21 | 56 | F | L3/4 | III | - | - | - | - | - | - | + + | + + | - |
| 22 | 78 | M | L5/S1 | III | - | - | - | - | - | - | + + | + + | + |
| 23 | 74 | M | L4/5 | III | - | - | - | - | - | - | + + | + + | + |
| 24 | 37 | F | L5/S1 | III | - | - | - | - | - | - | + + | + + | - |
| 25 | 56 | F | L4/5 | III | - | - | - | - | - | - | + + | + + | + |
| 26 | 60 | M | L4/5 | III | - | - | - | - | - | - | + + | + + | + |
| 27 | 58 | M | L5/S1 | III | - | - | - | - | - | - | + + | + + | + |
| 28 | 67 | M | L3/4 | III | - | - | - | - | - | - | + + | + + | + |
| 29 | 57 | M | L4/5 | III | - | - | - | - | - | - | + + | + + | + |
|  |  |  | L5/S1 | V | - | - | - | - | - | - | + + | + + | - |
| 30 | 40 | F | L4/5 | III | - | - | - | - | - | - | + + | + + | + |
| 31 | 55 | F | L4/5 | III | - | - | - | - | - | - | + + | + + | + |
| 32 | 47 | F | L4/5 | III | - | - | - | - | - | - | + + | + + | + |
| 33 | 59 | M | L4/5 | III | - | - | - | - | - | - | + + | + + | + |
| 34 | 71 | M | L4/5 | III | - | - | - | - | - | - | + + | + + | + |
| 35 | 49 | F | L4/5 | III | - | - | - | - | - | - | + + | + + | - |
| 36 | 42 | M | L4/5 | III | - | - | - | - | - | - | + + | + + | + |
| 37 | 40 | M | L5/S1 | III | - | - | - | - | - | - | + + | + + | + |
| 38 | 45 | M | L4/5 | III | - | - | - | - | - | - | + + | + + | + |
|  |  |  | L5/S1 | IV | - | - | - | - | - | - | + + | + + | + |
| 39 | 75 | M | L4/5 | III | - | - | - | - | - | - | + + | + + | + |
| 40 | 77 | M | L4/5 | III | - | - | - | - | - | - | + + | + + | - |
| 41 | 43 | F | L4/5 | III | - | - | - | - | - | - | + + | + + | - |
| 42 | 39 | M | L5/S1 | III | - | - | - | - | - | - | + + | + + | - |
| 43 | 58 | F | L4/5 | III | - | - | - | - | - | - | + + | + + | + |
| 44 | 34 | F | L4/5 | III | - | - | - | - | - | - | + + | + + | + |
| 45 | 64 | F | L5/S1 | III | - | - | - | - | - | - | + + | + + | + |
| 46 | 44 | M | L4/5 | III | - | - | - | - | - | - | + + | + + | - |
| (-)=positive cell is absent; (+)=positive cell number <20%; (+ +)= positive cell number >80% | | | | | | | | | | | | | |

| Case | Age | Gender | Site | Grade | CD20 | CD45RO | CD4 | CD8 | CD1a | S100 | CD68 | CD45 | CD11b |
| --- | --- | --- | --- | --- | --- | --- | --- | --- | --- | --- | --- | --- | --- |
| 47 | 48 | M | L4/5 | III | - | - | - | - | - | - | + + | + + | - |
| 48 | 41 | M | L3/4 | III | - | - | - | - | - | - | + + | + + | + |
| 49 | 39 | F | L3/4 | III | - | - | - | - | - | - | + + | + + | + |
|  |  |  | L4/5 | V | - | - | - | - | - | - | + + | + + | + |
| 50 | 55 | F | L3/4 | III | - | - | - | - | - | - | + + | + + | + |
| 51 | 50 | M | L5/S1 | III | - | - | - | - | - | - | + + | + + | - |
| 52 | 68 | M | L5/S1 | III | - | - | - | - | - | - | + + | + + | + |
| 53 | 71 | M | L5/S1 | III | - | - | - | - | - | - | + + | + + | + |
| 54 | 73 | F | L5/S1 | III | - | - | - | - | - | - | + + | + + | + |
| 55 | 84 | M | L3/4 | III | - | - | - | - | - | - | + + | + + | + |
| 56 | 43 | M | L5/S1 | III | - | - | - | - | - | - | + + | + + | + |
| 57 | 48 | F | L5/S1 | III | - | - | - | - | - | - | + + | + + | + |
| 58 | 67 | F | L5/S1 | III | - | - | - | - | - | - | + + | + + | + |
| 59 | 61 | F | L5/S1 | III | - | - | - | - | - | - | + + | + + | - |
| 60 | 66 | F | L4/5 | III | - | - | - | - | - | - | + + | + + | - |
| 61 | 66 | M | L4/5 | III | - | - | - | - | - | - | + + | + + | - |
|  |  |  | L5/S1 | IV | - | - | - | - | - | - | + + | + + | - |
| 62 | 58 | F | L5/S1 | III | - | - | - | - | - | - | + + | + + | - |
| 63 | 59 | M | L5/S1 | III | - | - | - | - | - | - | + + | + + | + |
| 64 | 54 | F | L4/5 | III | - | - | - | - | - | - | + + | + + | + |
| 65 | 63 | M | L4/5 | III | - | - | - | - | - | - | + + | + + | + |
| 66 | 69 | M | L4/5 | III | - | - | - | - | - | - | + + | + + | + |
| 67 | 68 | M | L5/S1 | III | - | - | - | - | - | - | + + | + + | + |
| 68 | 42 | F | L4/5 | III | - | - | - | - | - | - | + + | + + | + |
| 69 | 54 | F | L5/S1 | III | - | - | - | - | - | - | + + | + + | + |
| 70 | 56 | F | L5/S1 | III | - | - | - | - | - | - | + + | + + | - |
| 71 | 53 | F | L5/S1 | III | - | - | - | - | - | - | + + | + + | + |
| 72 | 65 | F | L5/S1 | III | - | - | - | - | - | - | + + | + + | + |
| 73 | 46 | M | L5/S1 | III | - | - | - | - | - | - | + + | + + | - |
| 74 | 69 | F | L5/S1 | III | - | - | - | - | - | - | + + | + + | + |
| 75 | 58 | M | L4/5 | III | - | - | - | - | - | - | + + | + + | + |
| 76 | 54 | F | L5/S1 | III | - | - | - | - | - | - | + + | + + | + |
| 77 | 51 | M | L5/S1 | III | - | - | - | - | - | - | + + | + + | - |
| 78 | 46 | F | L5/S1 | III | - | - | - | - | - | - | + + | + + | + |
| 79 | 59 | M | L3/4 | III | - | - | - | - | - | - | + + | + + | + |
| 80 | 32 | M | L4/5 | III | - | - | - | - | - | - | + + | + + | + |
| 81 | 48 | F | L5/S1 | III | - | - | - | - | - | - | + + | + + | + |
| 82 | 40 | F | L3/4 | III | - | - | - | - | - | - | + + | + + | + |
| 83 | 58 | M | L5/S1 | III | - | - | - | - | - | - | + + | + + | - |
| 84 | 68 | M | L3/4 | III | - | - | - | - | - | - | + + | + + | + |
| 85 | 42 | M | L4/5 | III | - | - | - | - | - | - | + + | + + | + |
|  |  |  | L5/S1 | V | - | - | - | - | - | - | + + | + + | + |
| 86 | 44 | F | L4/5 | III | - | - | - | - | - | - | + + | + + | - |
| 87 | 53 | M | L4/5 | III | - | - | - | - | - | - | + + | + + | + |
| 88 | 57 | F | L4/5 | III | - | - | - | - | - | - | + + | + + | - |
| 89 | 46 | F | L4/5 | III | - | - | - | - | - | - | + + | + + | + |
| 90 | 43 | M | L5/S1 | III | - | - | - | - | - | - | + + | + + | + |
| 91 | 40 | M | L4/5 | III | - | - | - | - | - | - | + + | + + | - |
|  |  |  | L5/S1 | V | - | - | - | - | - | - | + + | + + | - |
| 92 | 52 | M | L5/S1 | III | - | - | - | - | - | - | + + | + + | + |
| (-)=positive cell is absent; (+)=positive cell number <20%; (+ +)= positive cell number >80% | | | | | | | | | | | | | |

| Case | Age | Gender | Site | Grade | CD20 | CD45RO | CD4 | CD8 | CD1a | S100 | CD68 | CD45 | CD11b |
| --- | --- | --- | --- | --- | --- | --- | --- | --- | --- | --- | --- | --- | --- |
| 93 | 68 | F | L5/S1 | III | - | - | - | - | - | - | + + | + + | - |
| 94 | 53 | M | L3/4 | III | - | - | - | - | - | - | + + | + + | + |
|  |  |  | L4/5 | IV | - | - | - | - | - | - | + + | + + | + |
| 95 | 55 | M | L4/5 | III | - | - | - | - | - | - | + + | + + | + |
| 96 | 69 | M | L3/4 | III | - | - | - | - | - | - | + + | + + | + |
| 97 | 79 | M | L4/5 | III | - | - | - | - | - | - | + + | + + | + |
|  |  |  | L5/S1 | V | - | - | - | - | - | - | + + | + + | + |
| 98 | 81 | M | L3/4 | III | - | - | - | - | - | - | + + | + + | - |
| 99 | 77 | F | L4/5 | III | - | - | - | - | - | - | + + | + + | + |
| 100 | 39 | M | L5/S1 | III | - | - | - | - | - | - | + + | + + | - |
| 101 | 63 | F | L3/4 | III | - | - | - | - | - | - | + + | + + | + |
| 102 | 75 | M | L4/5 | III | - | - | - | - | - | - | + + | + + | - |
| 103 | 67 | F | L4/5 | III | - | - | - | - | - | - | + + | + + | + |
| 104 | 74 | M | L5/S1 | III | - | - | - | - | - | - | + + | + + | + |
| 105 | 40 | M | L5/S1 | III | - | - | - | - | - | - | + + | + + | + |
| 106 | 64 | M | L4/5 | III | - | - | - | - | - | - | + + | + + | - |
| 107 | 45 | M | L4/5 | IV | - | - | - | - | - | - | + + | + + | + |
| 108 | 54 | M | L4/5 | IV | - | - | - | - | - | - | + + | + + | - |
| 109 | 62 | F | L4/5 | IV | - | - | - | - | - | - | + + | + + | + |
| 110 | 55 | F | L4/5 | IV | - | - | - | - | - | - | + + | + + | - |
| 111 | 54 | F | L4/5 | IV | - | - | - | - | - | - | + + | + + | + |
| 112 | 46 | M | L4/5 | IV | - | - | - | - | - | - | + + | + + | + |
| 113 | 77 | M | L4/5 | IV | - | - | - | - | - | - | + + | + + | - |
| 114 | 68 | M | L4/5 | IV | - | - | - | - | - | - | + + | + + | + |
| 115 | 79 | M | L5/S1 | IV | - | - | - | - | - | - | + + | + + | + |
| 116 | 66 | F | L4/5 | IV | - | - | - | - | - | - | + + | + + | - |
| 117 | 61 | F | L4/5 | IV | - | - | - | - | - | - | + + | + + | - |
| 118 | 59 | F | L4/5 | IV | - | - | - | - | - | - | + + | + + | + |
| 119 | 48 | F | L4/5 | IV | - | - | - | - | - | - | + + | + + | + |
| 120 | 37 | F | L3/4 | IV | - | - | - | - | - | - | + + | + + | + |
| 121 | 59 | F | L5/S1 | IV | - | - | - | - | - | - | + + | + + | - |
| 122 | 38 | M | L5/S1 | IV | - | - | - | - | - | - | + + | + + | + |
| 123 | 43 | M | L4/5 | IV | - | - | - | - | - | - | + + | + + | + |
| 124 | 75 | F | L4/5 | IV | - | - | - | - | - | - | + + | + + | - |
| 125 | 78 | F | L4/5 | IV | - | - | - | - | - | - | + + | + + | + |
| 126 | 53 | M | L3/4 | IV | - | - | - | - | - | - | + + | + + | - |
| 127 | 74 | F | L5/S1 | IV | - | - | - | - | - | - | + + | + + | + |
| 128 | 65 | M | L3/4 | IV | - | - | - | - | - | - | + + | + + | + |
| 129 | 67 | F | L5/S1 | IV | - | - | - | - | - | - | + + | + + | + |
| 130 | 53 | F | L5/S1 | IV | - | - | - | - | - | - | + + | + + | + |
| 131 | 52 | M | L5/S1 | IV | - | - | - | - | - | - | + + | + + | + |
| 132 | 57 | M | L4/5 | IV | - | - | - | - | - | - | + + | + + | + |
| 133 | 66 | F | L4/5 | IV | - | - | - | - | - | - | + + | + + | - |
| 134 | 59 | M | L5/S1 | IV | - | - | - | - | - | - | + + | + + | + |
| 135 | 58 | F | L4/5 | IV | - | - | - | - | - | - | + + | + + | - |
|  |  |  | L5/S1 | V | - | - | - | - | - | - | + + | + + | - |
| 136 | 58 | M | L5/S1 | IV | - | - | - | - | - | - | + + | + + | + |
| 137 | 56 | F | L4/5 | IV | - | - | - | - | - | - | + + | + + | + |
| 138 | 72 | M | L4/5 | IV | - | - | - | - | - | - | + + | + + | + |
| 139 | 67 | M | L4/5 | IV | - | - | - | - | - | - | + + | + + | - |
| (-)=positive cell is absent; (+)=positive cell number <20%; (+ +)= positive cell number >80% | | | | | | | | | | | | | |

| Case | Age | Gender | Site | Grade | CD20 | CD45RO | CD4 | CD8 | CD1a | S100 | CD68 | CD45 | CD11b |
| --- | --- | --- | --- | --- | --- | --- | --- | --- | --- | --- | --- | --- | --- |
| 140 | 44 | M | L4/5 | IV | - | - | - | - | - | - | + + | + + | + |
| 141 | 49 | M | L5/S1 | IV | - | - | - | - | - | - | + + | + + | - |
| 142 | 44 | M | L3/4 | IV | - | - | - | - | - | - | + + | + + | - |
|  |  |  | L5/S1 | V | - | - | - | - | - | - | + + | + + | - |
| 143 | 58 | FF | L5/S1 | IV | - | - | - | - | - | - | + + | + + | + |
| 144 | 54 | F | L3/4 | IV | - | - | - | - | - | - | + + | + + | + |
| 145 | 46 | M | L4/5 | IV | - | - | - | - | - | - | + + | + + | + |
| 146 | 46 | M | L4/5 | IV | - | - | - | - | - | - | + + | + + | + |
| 147 | 53 | F | L4/5 | IV | - | - | - | - | - | - | + + | + + | - |
| 148 | 78 | M | L4/5 | IV | - | - | - | - | - | - | + + | + + | - |
| 149 | 72 | M | L5/S1 | IV | - | - | - | - | - | - | + + | + + | + |
| 150 | 59 | F | L5/S1 | IV | - | - | - | - | - | - | + + | + + | + |
| 151 | 78 | M | L4/5 | IV | - | - | - | - | - | - | + + | + + | + |
| 152 | 48 | F | L5/S1 | IV | - | - | - | - | - | - | + + | + + | + |
| 153 | 68 | M | L5/S1 | IV | - | - | - | - | - | - | + + | + + | + |
| 154 | 74 | F | L4/5 | IV | - | - | - | - | - | - | + + | + + | + |
| 155 | 62 | M | L5/S1 | IV | - | - | - | - | - | - | + + | + + | + |
| 156 | 74 | M | L5/S1 | IV | - | - | - | - | - | - | + + | + + | + |
|  |  |  | L5/S1 | IV | - | - | - | - | - | - | + + | + + | + |
| 157 | 44 | M | L4/5 | IV | - | - | - | - | - | - | + + | + + | - |
| 158 | 64 | F | L4/5 | IV | - | - | - | - | - | - | + + | + + | - |
| 159 | 72 | F | L3/4 | IV | - | - | - | - | - | - | + + | + + | + |
| 160 | 34 | M | L4/5 | IV | - | - | - | - | - | - | + + | + + | + |
| 161 | 78 | M | L4/5 | IV | - | - | - | - | - | - | + + | + + | + |
|  |  |  | L4/5 | IV | - | - | - | - | - | - | + + | + + | - |
| 162 | 71 | M | L5/S1 | IV | - | - | - | - | - | - | + + | + + | - |
| 163 | 43 | M | L4/5 | IV | - | - | - | - | - | - | + + | + + | + |
| 164 | 67 | M | L3/4 | IV | - | - | - | - | - | - | + + | + + | - |
| 165 | 66 | F | L5/S1 | IV | - | - | - | - | - | - | + + | + + | + |
| 166 | 56 | M | L5/S1 | IV | - | - | - | - | - | - | + + | + + | + |
| 167 | 65 | F | L5/S1 | IV | - | - | - | - | - | - | + + | + + | - |
| 168 | 51 | F | L4/5 | IV | - | - | - | - | - | - | + + | + + | + |
|  |  |  | L5/S1 | V | - | - | - | - | - | - | + + | + + | + |
| 169 | 64 | F | L4/5 | IV | - | - | - | - | - | - | + + | + + | - |
| 170 | 58 | M | L4/5 | IV | - | - | - | - | - | - | + + | + + | + |
| 171 | 57 | M | L5/S1 | IV | - | - | - | - | - | - | + + | + + | + |
| 172 | 53 | M | L5/S1 | IV | - | - | - | - | - | - | + + | + + | + |
| 173 | 58 | M | L5/S1 | IV | - | - | - | - | - | - | + + | + + | + |
| 174 | 45 | F | L3/4 | IV | - | - | - | - | - | - | + + | + + | + |
| 175 | 73 | M | L5/S1 | IV | - | - | - | - | - | - | + + | + + | + |
| 176 | 56 | F | L4/5 | IV | - | - | - | - | - | - | + + | + + | + |
| 177 | 58 | M | L5/S1 | IV | - | - | - | - | - | - | + + | + + | + |
| 178 | 47 | M | L3/4 | IV | - | - | - | - | - | - | + + | + + | - |
|  |  |  | L4/5 | V | - | - | - | - | - | - | + + | + + | - |
| 179 | 43 | M | L5/S1 | IV | - | - | - | - | - | - | + + | + + | + |
| 180 | 39 | M | L5/S1 | IV | - | - | - | - | - | - | + + | + + | + |
| 181 | 29 | M | L5/S1 | IV | - | - | - | - | - | - | + + | + + | + |
| 182 | 40 | F | L5/S1 | IV | - | - | - | - | - | - | + + | + + | + |
| 183 | 64 | M | L5/S1 | IV | - | - | - | - | - | - | + + | + + | + |
| 184 | 55 | F | L4/5 | IV | - | - | - | - | - | - | + + | + + | - |
| 185 | 50 | F | L4/5 | IV | - | - | - | - | - | - | + + | + + | + |
| 186 | 49 | M | L5/S1 | IV | - | - | - | - | - | - | + + | + + | + |
| (-)=positive cell is absent; (+)=positive cell number <20%; (+ +)= positive cell number >80% | | | | | | | | | | | | | |

| Case | Age | Gender | Site | Grade | CD20 | CD45RO | CD4 | CD8 | CD1a | S100 | CD68 | CD45 | CD11b |
| --- | --- | --- | --- | --- | --- | --- | --- | --- | --- | --- | --- | --- | --- |
| 187 | 49 | F | L5/S1 | IV | - | - | - | - | - | - | + + | + + | + |
|  |  |  | L5/S1 | V | - | - | - | - | - | - | + + | + + | + |
| 188 | 79 | F | L5/S1 | IV | - | - | - | - | - | - | + + | + + | - |
| 189 | 56 | M | L3/4 | IV | - | - | - | - | - | - | + + | + + | - |
| 190 | 81 | F | L5/S1 | IV | - | - | - | - | - | - | + + | + + | - |
| 191 | 62 | M | L5/S1 | IV | - | - | - | - | - | - | + + | + + | + |
| 192 | 58 | M | L5/S1 | IV | - | - | - | - | - | - | + + | + + | + |
| 193 | 66 | M | L4/5 | IV | - | - | - | - | - | - | + + | + + | - |
|  |  |  | L5/S1 | V | - | - | - | - | - | - | + + | + + | + |
| 194 | 54 | M | L4/5 | IV | - | - | - | - | - | - | + + | + + | + |
| 195 | 67 | M | L5/S1 | IV | - | - | - | - | - | - | + + | + + | + |
| 196 | 72 | F | L5/S1 | IV | - | - | - | - | - | - | + + | + + | - |
| 197 | 87 | F | L5/S1 | IV | - | - | - | - | - | - | + + | + + | + |
| 198 | 40 | F | L4/5 | IV | - | - | - | - | - | - | + + | + + | + |
| 199 | 71 | M | L4/5 | IV | - | - | - | - | - | - | + + | + + | - |
|  |  |  | L4/5 | IV | - | - | - | - | - | - | + + | + + | + |
| 200 | 40 | M | L4/5 | IV | - | - | - | - | - | - | + + | + + | + |
| 201 | 43 | M | L4/5 | IV | - | - | - | - | - | - | + + | + + | - |
|  |  |  | L5/S1 | V | - | - | - | - | - | - | + + | + + | + |
| 202 | 46 | F | L5/S1 | IV | - | - | - | - | - | - | + + | + + | + |
| 203 | 47 | M | L5/S1 | IV | - | - | - | - | - | - | + + | + + | - |
| 204 | 49 | F | L5/S1 | IV | - | - | - | - | - | - | + + | + + | + |
| 205 | 85 | M | L5/S1 | IV | - | - | - | - | - | - | + + | + + | + |
| 206 | 56 | F | L3/4 | IV | - | - | - | - | - | - | + + | + + | + |
| 207 | 55 | M | L5/S1 | IV | - | - | - | - | - | - | + + | + + | + |
| 208 | 53 | M | L3/4 | IV | - | - | - | - | - | - | + + | + + | + |
| 209 | 82 | M | L3/4 | IV | - | - | - | - | - | - | + + | + + | + |
| 210 | 58 | F | L4/5 | IV | - | - | - | - | - | - | + + | + + | - |
| 211 | 69 | F | L5/S1 | V | - | - | - | - | - | - | + + | + + | - |
| 212 | 59 | M | L5/S1 | V | - | - | - | - | - | - | + + | + + | + |
| 213 | 81 | F | L4/5 | V | - | - | - | - | - | - | + + | + + | + |
|  |  |  | L5/S1 | V | - | - | - | - | - | - | + + | + + | - |
| 214 | 61 | F | L5/S1 | V | - | - | - | - | - | - | + + | + + | + |
| 215 | 63 | F | L5/S1 | V | - | - | - | - | - | - | + + | + + | + |
| 216 | 76 | M | L4/5 | V | - | - | - | - | - | - | + + | + + | + |
| 217 | 54 | M | L5/S1 | V | - | - | - | - | - | - | + + | + + | - |
| 218 | 47 | F | L4/5 | V | - | - | - | - | - | - | + + | + + | + |
| 219 | 49 | M | L5/S1 | V | - | - | - | - | - | - | + + | + + | - |
| 220 | 46 | M | L5/S1 | V | - | - | - | - | - | - | + + | + + | + |
| 221 | 56 | M | L5/S1 | V | - | - | - | - | - | - | + + | + + | + |
| 222 | 76 | M | L4/5 | V | - | - | - | - | - | - | + + | + + | - |
| 223 | 43 | M | L5/S1 | V | - | - | - | - | - | - | + + | + + | + |
| 224 | 77 | F | L5/S1 | V | - | - | - | - | - | - | + + | + + | - |
| 225 | 55 | F | L4/5 | V | - | - | - | - | - | - | + + | + + | + |
| 226 | 49 | F | L5/S1 | V | - | - | - | - | - | - | + + | + + | + |
| 227 | 71 | M | L5/S1 | V | - | - | - | - | - | - | + + | + + | + |
| 228 | 50 | M | L5/S1 | V | - | - | - | - | - | - | + + | + + | - |
| 229 | 60 | M | L5/S1 | V | - | - | - | - | - | - | + + | + + | + |
| 230 | 45 | M | L4/5 | V | - | - | - | - | - | - | + + | + + | + |
| 231 | 47 | M | L4/5 | V | - | - | - | - | - | - | + + | + + | - |
| (-)=positive cell is absent; (+)=positive cell number <20%; (+ +)= positive cell number >80% | | | | | | | | | | | | | |

| Case | Age | Gender | Site | Grade | CD20 | CD45RO | CD4 | CD8 | CD1a | S100 | CD68 | CD45 | CD11b |
| --- | --- | --- | --- | --- | --- | --- | --- | --- | --- | --- | --- | --- | --- |
| 232 | 84 | M | L5/S1 | V | - | - | - | - | - | - | + + | + + | + |
| 233 | 73 | F | L5/S1 | V | - | - | - | - | - | - | + + | + + | - |
| 234 | 56 | M | L5/S1 | V | - | - | - | - | - | - | + + | + + | + |
| 235 | 44 | F | L4/5 | V | - | - | - | - | - | - | + + | + + | - |
| 236 | 45 | F | L5/S1 | V | - | - | - | - | - | - | + + | + + | + |
| 237 | 54 | F | L5/S1 | V | - | - | - | - | - | - | + + | + + | + |
| 238 | 68 | F | L5/S1 | V | - | - | - | - | - | - | + + | + + | - |
| 239 | 63 | M | L5/S1 | V | - | - | - | - | - | - | + + | + + | + |
| 240 | 67 | M | L5/S1 | V | - | - | - | - | - | - | + + | + + | + |
| 241 | 75 | F | L5/S1 | V | - | - | - | - | - | - | + + | + + | + |
| 242 | 45 | F | L5/S1 | V | - | - | - | - | - | - | + + | + + | - |
| 243 | 67 | M | L5/S1 | V | - | - | - | - | - | - | + + | + + | + |
| 244 | 55 | M | L5/S1 | V | - | - | - | - | - | - | + + | + + | + |
| 245 | 54 | F | L5/S1 | V | - | - | - | - | - | - | + + | + + | - |
| 246 | 57 | M | L5/S1 | V | - | - | - | - | - | - | + + | + + | - |
| 247 | 63 | F | L5/S1 | V | - | - | - | - | - | - | + + | + + | + |
| 248 | 59 | M | L5/S1 | V | - | - | - | - | - | - | + + | + + | + |
| 249 | 60 | M | L5/S1 | V | - | - | - | - | - | - | + + | + + | + |
| 250 | 64 | M | L4/5 | V | - | - | - | - | - | - | + + | + + | + |
| 251 | 53 | F | L5/S1 | V | - | - | - | - | - | - | + + | + + | + |
| 252 | 47 | F | L5/S1 | V | - | - | - | - | - | - | + + | + + | - |
| 253 | 68 | F | L5/S1 | V | - | - | - | - | - | - | + + | + + | + |
| 254 | 59 | F | L5/S1 | V | - | - | - | - | - | - | + + | + + | + |
| 255 | 49 | M | L5/S1 | V | - | - | - | - | - | - | + + | + + | + |
| 256 | 51 | F | L5/S1 | V | - | - | - | - | - | - | + + | + + | + |
| 257 | 59 | F | L5/S1 | V | - | - | - | - | - | - | + + | + + | + |
| 258 | 67 | M | L5/S1 | V | - | - | - | - | - | - | + + | + + | - |
| 259 | 63 | M | L5/S1 | V | - | - | - | - | - | - | + + | + + | + |
| 260 | 76 | M | L5/S1 | V | - | - | - | - | - | - | + + | + + | + |
| 261 | 56 | M | L4/5 | V | - | - | - | - | - | - | + + | + + | - |
| 262 | 55 | M | L5/S1 | V | - | - | - | - | - | - | + + | + + | + |
| 263 | 49 | M | L5/S1 | V | - | - | - | - | - | - | + + | + + | + |
| 264 | 45 | M | L5/S1 | V | - | - | - | - | - | - | + + | + + | + |
| 265 | 51 | M | L5/S1 | V | - | - | - | - | - | - | + + | + + | - |
| 266 | 59 | M | L5/S1 | V | - | - | - | - | - | - | + + | + + | + |
| 267 | 41 | F | L4/5 | V | - | - | - | - | - | - | + + | + + | + |
| 268 | 69 | M | L5/S1 | V | - | - | - | - | - | - | + + | + + | + |
| 269 | 77 | F | L4/5 | V | - | - | - | - | - | - | + + | + + | - |
| 270 | 61 | M | L4/5 | V | - | - | - | - | - | - | + + | + + | + |
| 271 | 54 | F | L5/S1 | V | - | - | - | - | - | - | + + | + + | + |
| 272 | 64 | M | L5/S1 | V | - | - | - | - | - | - | + + | + + | - |
| 273 | 43 | F | L5/S1 | V | - | - | - | - | - | - | + + | + + | + |
| 274 | 56 | M | L4/5 | V | - | - | - | - | - | - | + + | + + | + |
|  |  |  | L5/S1 | V | - | - | - | - | - | - | + + | + + | + |
| 275 | 73 | F | L4/5 | V | - | - | - | - | - | - | + + | + + | - |
| 276 | 44 | M | L5/S1 | V | - | - | - | - | - | - | + + | + + | + |
| 277 | 71 | M | L5/S1 | V | - | - | - | - | - | - | + + | + + | + |
| 278 | 69 | M | L5/S1 | V | - | - | - | - | - | - | + + | + + | - |
| (-)=positive cell is absent; (+)=positive cell number <20%; (+ +)= positive cell number >80% | | | | | | | | | | | | | |

**TableS2.** Primers used in Q–PCR

| \| Name­­­­ \| Sequence \| \| --- \| --- \| \| P38α \| 5′-CCATGAGGCAAGAAACTA -3′  5′-AAACGAGTCATGGTGCTA -3′ \| \| P38β \| 5′-AGAAGGTGGCGGTGAAGAA-3′  5′-GCAGTAGCCCGAAGACCT -3′ \| \| P38γ \| 5′-TGCCTCTATCCTGACCAATG -3′  5′-GCTGTGCCTTCTACTCGG -3′ \| \| P38δ \| 5′-CTCACCCATCCCTTCTTTG-3′  5′-CTACTTACCTTCGTCGTGT -3′ \| \| ADAMTS-4 \| 5′-GGGATAGTGACCACATTGTT-3′  5′-AGGCACTGGGCTACTACTAT-3′ \| \| ADAMTS-5 \| 5′-AGGCACTGGGCTACTACTAT-3′  5′-CGCTTATCTTCTGTGGAACCAAA-3′ \| \| MMP-13 \| 5′-CGACTTCTACCCATTTGA -3′  5′-ACTTTGGACCTGTTCATCA -3′ \| \| CCL3 \| 5′-CCTTGCTGTCCTCCTCTG -3′  5′-AACTCTGCTCGTCGGTC -3′ \| \| CoL-II \| 5′-TGGTGGCTTCCATTTCAGCT -3′  5′-TGTTCTGGGAGCCTTCCGT -3′ \| \| Aggrecan \| 5′-AGCCTGCGCTCCAATGACT -3′  5′-GGAACACGATGCCTTTCACC-3′ \| \| β-actin \| 5′-GCATGGGTCAGAAGGATTCCT -3′  5′-TCGTCCCAGTTGGTGACGAT -3′ \| |
| --- | --- | --- | --- | --- | --- | --- | --- | --- | --- | --- | --- | --- | --- | --- | --- | --- | --- | --- | --- | --- | --- | --- | --- | --- |
